# A rare presentation of secondary multiple miliary osteoma cutis

**DOI:** 10.1016/j.jdcr.2023.01.010

**Published:** 2023-01-30

**Authors:** Ana Duarte-Summers, Anna Bistline, Viral Patel, Elizabeth Jones

**Affiliations:** a Thomas Jefferson University Sidney Kimmel Medical College, Philadelphia, Pennsylvania; b Thomas Jefferson University Hospital, Philadelphia, Pennsylvania

**Keywords:** acne, calcification, calcinosis cutis, cutaneous calcification, dystrophic calcinosis cutis, multiple miliary osteoma cutis, nodules, osteoma cutis, radiation, secondary osteoma cutis, truncal osteoma cutis, MMOC, multiple miliary osteoma cutis, SOC, secondary osteoma cutis

An 80-year-old male presented with firm pruritic skin-colored to yellow papules and nodules on his neck, chest, and back present for decades (Figs 1 and 2). In adolescence, he received radiation therapy for severe acne. Medications include emtricitabine and tenofovir alafenamide for HIV. As the appearance of the lesions preceded initiation of emtricitabine and tenofovir alafenamide by several years, the cutaneous findings were concluded to be unrelated to these medications. Serum calcium, alkaline phosphatase, and complete blood cell count were normal. The patient did not have other medical conditions or family history of similar cutaneous findings. A shave biopsy of a papule from the chest demonstrated spicules of bone within the dermis with dense calcification and overlying epidermal ulceration (Fig 3). Based on clinicopathologic correlation, the patient was diagnosed with multiple miliary osteoma cutis with findings of secondary calcification.

**Fig 1:**
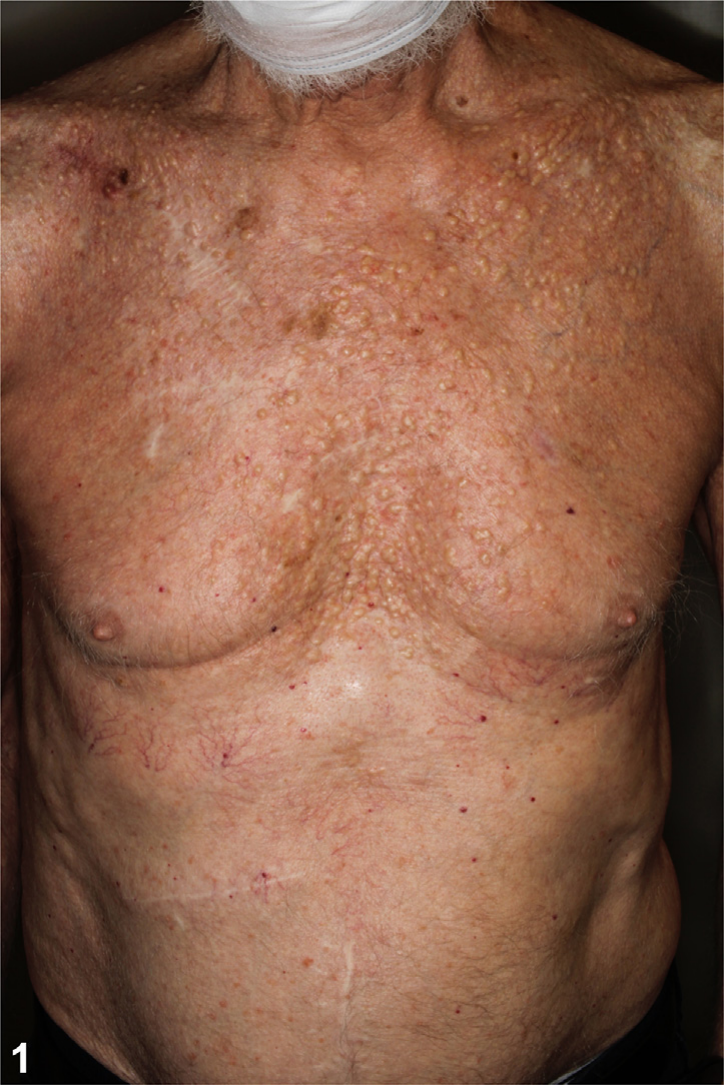

**Fig 2:**
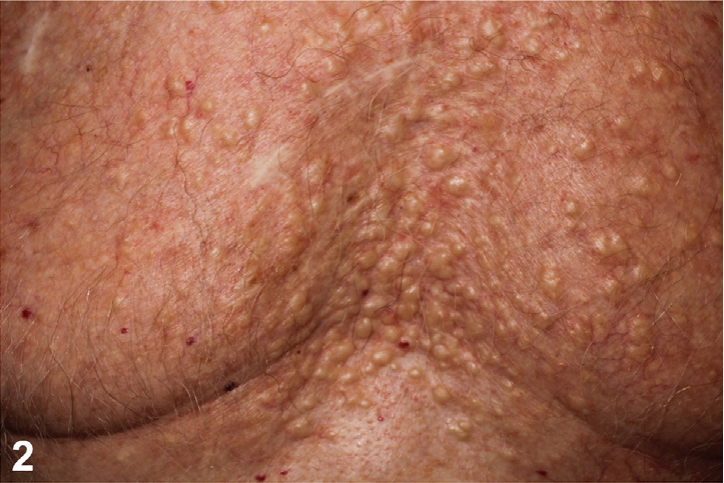

**Fig 3:**
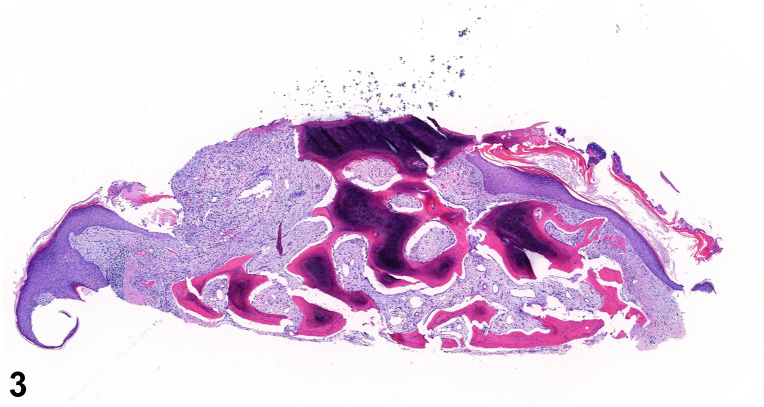

**Question 1: What is the most typical presentation of secondary multiple miliary osteoma cutis?**

A. Male patient, lesions on the face
B. Female patient, lesions on the face
C. Male patient, lesions on the trunk
D. Female patient, lesions on the trunk
E. Monomorphous pink papules on the face and trunk

**Answers:**

A. Male patient, lesions on the face – Incorrect. Secondary osteoma cutis (SOC) is the metaplastic development of bone tissue associated with a predisposing factor such as inflammation, or neoplastic changes that lead to degradation of collagen fibers, such as a history of severe and prolonged inflammation associated with severe acne.^1^ Secondary multiple miliary osteoma cutis (MMOC) typically occurs on the face and tends to affect middle-aged women with a sex ratio of 8:1.^2^ A global search by Duarte and Pinheiro spanning from 1926 to 2017 identified 102 reported cases of secondary MMOC. Of these cases, only 11% were male, 12% affected the thorax, and only 3.6% involved the back.^2^
B. Female patient, lesions on the face – Correct. Secondary MMOC typically occurs on the face and tends to affect middle-aged women with a sex ratio of 8:1.^2^
C. Male patient, lesions on the trunk – Incorrect. The condition in men more commonly involves the neck or thorax, however, this is not the most common presentation of Secondary MMOC.^3^
D. Female patient, lesions on the trunk – Incorrect. Secondary MMOC commonly presents on the face in women. In men the condition more commonly involves the trunk.^3^
E. Monomorphous pink papules on the face and trunk – Incorrect. Monomorphous pink papules on the face and trunk are commonly associated with steroid-induced acne.

**Question 2: What is the primary predisposing favor to developing secondary osteoma cutis?**

A. History of exposure to radiation
B. History of exposure to steroids
C. History of exposure to isotretinoin therapy
D. History of inflammatory acne
E. History of pruritus and excoriation

**Answers:**

A. History of exposure to radiation – Incorrect. While rare cases have posited that a history of radiation therapy may have led to SOC decades later, MMOC is more commonly associated with a history of severe and prolonged inflammation, such as prolonged inflammation and trauma associated with severe acne.^1^ A history of radiation therapy for various types of cancers has been described in association with dystrophic calcinosis cutis.^4^ Dystrophic calcinosis cutis was an important differential diagnosis given this patient’s history of acne and radiation treatment. Dystrophic calcinosis cutis has been associated with trauma to the skin, including acne, burns, chronic inflammation, various connective tissue disorders, and a history of radiation therapy.^4^ While similar in clinical presentation, osteoma cutis is the ossification of the dermis and subcutaneous tissue, whereas dystrophic calcinosis cutis is the deposition of insoluble calcium salts in tissues.^4^
B. History of exposure to steroids – Incorrect. Exposure to steroids has not commonly been reported in association with MMOC; 85% of cases of SOC are thought to be caused by severe acne, but not necessarily directly attributable to use of steroids.^3^
C. History of exposure to isotretinoin therapy – Incorrect. While there are case studies linking isotretinoin therapy to exacerbation of SOC,^5^ isotretinoin therapy is not the primary predisposing factor to developing SOC.
D. History of inflammatory acne – Correct. Acne is thought to be responsible for 85% of SOC.^3^ Several cases report SOC presenting as MMOC arising decades after acne.^1^
E. History of pruritus and excoriation – Incorrect. Pruritis and excoriation has been reported in association with formation of osteoma cutis, but this is rare.^1^ Pruritis and excoriation are not primary predisposing factors to developing SOC.

**Question 3: A biopsy of osteoma cutis will likely feature which of the following?**

A. Eosinophilic spicules with osteocytes, cempent lines, and focal calcification
B. Dense basophilic deposits within the dermis accompanied by a granulomatous reaction
C. Cystic structure lined by a serpiginous eosinophilic cuticle and sebaceous glands
D. Mixture of basophilic cells and eosinophilic “ghost” like cells, calcium deposits and a granulomatous reaction
E. Cystic structure lined by stratified squamous epithelium with a granular layer

**Answers:**

A. Eosinophilic spicules with osteocytes, cempent lines, and focal calcification – Correct. Osteoma cutis typically presents as eosinophilic spicules with osteocytes, cement lines, and focal calcification on histology. The shave biopsies above show numerous osteocytes within small lacunae and several cement lines. The stroma is fibrovascular without any bone marrow elements.
B. Dense basophilic deposits within the dermis accompanied by a granulomatous reaction – Incorrect. This is a classic pathology description for calcinosis cutis.
C. Cystic structure lined by a serpiginous eosinophilic cuticle and sebaceous glands – Incorrect. This is a classic pathology description for steatocystomas.
D. Mixture of basophilic cells and eosinophilic “ghost” like cells, calcium deposits and a granulomatous reaction – Incorrect. This is a classic pathology description for pilomatricomas.
E. Cystic structure lined by stratified squamous epithelium with a granular layer – Incorrect. This is a classic pathology description for epidermal cysts.

## Conflicts of interest

None disclosed.

## References

[1] Niebel D., Poortinga S., Wenzel J. (2020). Osteoma cutis and calcinosis cutis: “similar but different”. J Clin Aesthet Dermatol.

[2] Duarte B.M., Pinheiro R.R., Cabete J. (2018). Multiple miliary osteoma cutis: a comprehensive review and update of the literature. Eur J Dermatol.

[3] Alhazmi D., Badr F., Jadu F., Jan A.M., Abdulsalam Z. (2017). Osteoma cutis of the face in CBCT images. Case Rep Dent.

[4] Perna D., Margheim A., Schadt C.R. (2021). Radiation-induced morphea and dystrophic calcinosis cutis of the breast. Int J Dermatol.

[5] Brodkin R.H., Abbey A.A. (1985). Osteoma cutis: a case of probable exacerbation following treatment of severe acne with isotretinoin. Dermatology.

